# How to make a difference: the impact of gender-fair language on text comprehensibility amongst adults with and without an academic background

**DOI:** 10.3389/fpsyg.2023.1234860

**Published:** 2023-12-15

**Authors:** Laura Mathilde Pabst, Marlene Kollmayer

**Affiliations:** Department of Developmental and Educational Psychology, Faculty of Psychology, University of Vienna, Vienna, Austria

**Keywords:** gender-fair language, masculine generics, grammatical gender, text comprehensibility, adults, academic background

## Abstract

**Introduction:**

The proliferation of gender-fair language as a medium of communication that represents all genders can be considered as an exciting development in today's rapidly changing world. In this context, the use of the gender asterisk has become especially prominent in German, it being a grammatical gender language. However, critics often argue that gender-fair language makes texts less comprehensible and decreases its aesthetic appeal. The present study tests this assumption for the German language and is the first one to test the influence of an academic background on the comprehensibility of gender-fair language.

**Method:**

A text, either written in gender-fair language using the gender star in its singular and plural form or a version using only masculine-only forms, was randomly assigned to 81 adults without an academic background and 82 adults with an academic background (77% women in both groups). Participants were asked to fill out a web-based questionnaire answering questions on text comprehensibility and on their attitudes toward gender-fair language.

**Results:**

The results show no statistically significant difference in comprehensibility ratings between participants who read a text in gender-fair language and those who read a text in masculine-only language. In addition, attitudes toward gender-fair language did not affect comprehensibility ratings in participants who read the text written in gender-fair language using the gender star. Further, the academic background had no effect on the assessment of gender-fair language.

**Discussion:**

To conclude, the present study suggests that there is no evidence that gender-fair language reduces the comprehensibility of texts.

## 1 Introduction

At the beginning of the 1970s, the question whether texts should be written using masculine-only-forms or applying gender-fair language has first been raised (Braun et al., 2005). The use of grammatically masculine-only forms to refer to both male and female exemplars, also considered as masculine generics, has often been criticized for predominantly evoking mental images of men and thus triggering a male bias (Gabriel et al., 2008). This linguistic gender asymmetry can be considered as omnipresent as it is ubiquitous in a large variety of languages (Hellinger and Bußmann, 2001/2002/2003). An example is the German masculine generic *Lehrer*, “teachers (male),” which is interpreted to refer to both *Lehrer* (m. pl.) and *Lehrerinnen* (f. pl.). Contrary to masculine-only forms, feminine-only forms do not function in the same way as they are usually used to refer to women only (Hellinger and Bußmann, 2001/2002/2003). Various studies have confirmed that, by decreasing the visibility of women and thereby limiting the cognitive accessibility of females, existing gender inequalities may be reinforced (Keith et al., 2022). Actually, Körner et al. (2022) have recently conducted two experiments in which they analyzed gender representations that had been activated while reading sentences in the gender star form in comparison to the masculine-only and pair forms: The findings showed that after encountering the masculine-only form, people tended to judge continuations about men more frequently and quickly, indicating a male bias, even when they were informed about the generic intention.

In contrast to the generic masculine, gender-fair language not only aims at increasing the visibility of women, but rather intends to include all genders (Kolek, 2019). Its goal is to reduce discrimination and gender stereotyping (Sczesny et al., 2016). In recent times, research on gender-fair language has actually boomed. Inter alia, a special focus has been put on the links between gender-fair language and gendered occupational beliefs (Vervecken et al., 2015; Horvath et al., 2016). Since critics have argued that text comprehension and the text's aesthetic appeal would be impaired through the use of gender-fair language (e.g., Braun et al., 2007; Stahlberg et al., 2007), another key research field investigating the effects of gender-fair language on the comprehensibility of texts has been established.

Instead, though, findings seem to paint a rather clear picture and are consistent in the results that gender-fair language did not impair comprehensibility. However, different effects for various forms of gender-fair language in German, said language being the focus of the present study, could have been shown. Overall, these various forms of gender-fair language are as follows:

1) pair forms: e.g., *Leserin oder Leser*, “reader (female) or reader (masculine)”
2) neutral forms: e.g., *Lesende*, ≈ “those who read”
3) capital-I forms: e.g., *LeserIn*, ≈ “feMale reader”
4) gender asterisk in plural forms: e.g., Leser^*^innen, ≈ “the fe^*^male readers”
5) gender asterisks in singular forms: e.g., Leser^*^in, ≈ “the fe^*^male reader”
6) slashes: e.g., *Leser/in*, ≈ “fe/male reader”.

To be more precise, previous studies indicated that (1) pair forms (e.g., Braun et al., 2007; Friedrich and Heise, 2019), (2) neutral forms (e.g., Rothmund and Christmann, 2002; Steiger-Loerbroks and von Stockhausen, 2014), (3) capital-I forms (e.g., Klimmt et al., 2008; Pöschko and Prieler, 2018) and (4) gender asterisks in plural forms (e.g., Friedrich et al., 2021) did not negatively affect comprehensibility. In contrast, gender asterisks in singular forms and slashes did so (Friedrich et al., 2021). To elaborate on these somewhat ambiguous results of Friedrich et al. (2021), the study's design as well as the results are first being presented in more detail:

To start off, the researchers employed a between-subjects design, focusing on the variable of language form in two experiments. In the first experiment, no statistically relevant impairing effects of gender-fair language on text comprehension or interest in the game were discovered. The variable interest in the game was included to investigate the generalisability of findings on the impact of gender-fair language on interest and commitment in a vocational setting to other domains. In their second experiment, however, comprehensibility, aesthetic evaluation, and interest in the game were significantly impaired when the instructions used gender-fair language. The reasons for this ambiguity are difficult to determine as several variables–such as the used texts and the adaptation to gender-fair language in terms of the quantity of the manipulated passages–have been manipulated simultaneously.

At this point, it is important to note that creating gender-fair language in German is typically less challenging for plural forms compared to singular forms. This is probably because there is only one article for the plural form in German (*die*), but there are three different forms in the singular (*der, die, das*).

Another limitation in the research of the field of gender-fair language in general refers to the fact that the majority of the studies' samples mainly consist of university students in the educational, psychological, and medical sectors. It can be inferred that the individuals examined have greater familiarity with the gender asterisk compared to other groups and hold a favorable stance toward the use of gender-inclusive language (Friedrich et al., 2021). This is why, the present study pursues the question of whether the use of the gender asterisk as a form of gender-fair language has an impact on text comprehensibility in academics and non-academics.

### 1.1 Language structures and strategies for gender-fair language

Generally speaking, there are various ways how languages represent gender. Stahlberg et al. (2007) suggest the following distinction that has found widespread use in research: (1) grammatical gender languages, (2) natural gender languages and (3) genderless languages. In addition, Gygax et al. (2019, p. 4) have added two further language groups, namely “[l]anguages with a combination of grammatical gender and natural gender (e.g., Norwegian, Dutch)” and “[g]enderless languages with a few traces of grammatical gender (e.g., Oriya, Basque).” Grammatical gender languages such as German or French are characterized by each noun having a fixed grammatical gender; further, personal nouns usually reflect the gender of the entity they are referring to Sczesny et al. (2016) (e.g., German *Lehrer*_*masc*_/*Lehrerin*_*fem*_, “male/female teacher,” French *professeur*_*masc*_/*professeure*_*fem*_, “male/female teacher”) and personal pronouns carry information on the respective gender (e.g., German *er*/*sie*, “he/she”). Natural gender languages such as English or Swedish mostly contain personal nouns that are gender-neutral (e.g., English “teacher,” “writer”); still, there are personal pronouns that differentiate gender (e.g., Swedish *han*/*hon*, English “he/she”) (Sczesny et al., 2016). Lastly, genderless languages such as Finnish or Turkish can only express gender by using attributes [e.g., “male/female (teacher)”] or lexical gender nouns (e.g., “mother,” “father”); not only do personal nouns not denote gender, even pronouns do not differentiate for gender (Sczesny et al., 2016).

In order to avoid the detrimental effects of masculine-only forms, the question must be raised which strategies can be applied to make language more gender-fair. In general, there are two common strategies to make language more inclusive, namely neutralization and feminisation (Gabriel et al., 2018). Overall, neutralization tries to avoid gender markings and is most often used in natural gender languages (Sczesny et al., 2016). In contrast, feminisation aims at enhancing the visibility of female exemplars by explicitly referring to them (Stormbom, 2019). This is actually a crucial difference between the two mentioned strategies: Feminisation directly activates a binary categorization of gender by naming the feminine and masculine forms, whereas neutralization tries to overcome this outdated concept (Gabriel et al., 2018). “Broadly, [neutralization] refers to the idea of abandoning the explicit mention of female or male gender” (Gabriel et al., 2018, p. 850). Instead, it uses gender indefinite nouns. For example, “steward” and “stewardess” are being replaced by “flight attendant.”

Feminisation is most often used to make grammatical gender languages gender-fair (Sczesny et al., 2016). For example, instead of only using the German term *Lehrer* (“teachers”) to refer to both men and women, one could instead use *Lehrerinnen und Lehrer* (“teachers_fem_” and “teachers_masc_”). Moreover, in recent times, the use of the gender asterisk (“Gendersternchen”) has become more prevalent in languages that have grammatical genders as a means of representing individuals of all genders, e.g., Lehrer^*^innen (“teachers of all genders”) (Diewald and Steinhauer, 2020). The gender asterisk aims at explicitly including non-binary individuals and challenging the gender binary.

### 1.2 Consequences of gender-exclusive and gender-fair language use

It is not surprising that especially grammatical gender languages are prone to gender and linguistic inequalities. This hypothesis is supported by Prewitt-Freilino et al. (2012) who used the *Global Gender Gap Index* of the *World Economic Forum* to find out whether there are differences in gender equality regarding rights, responsibilities and opportunities that are associated with linguistics. According to their research results, the use of gendered languages is associated with lower levels of gender equality in a language community. In line with this, language communities that use natural gender languages tend to exhibit higher levels of gender equality than those with grammatical gender languages (Hausmann et al., 2009). Prewitt-Freilino et al. (2012) attributed this finding to the fact that it is easier to make gender-neutral modifications to instances of sexist language in natural gender languages than in grammatical gender languages that depend upon gendered structures in total. While this causal attribution is a rather strong claim derived from a correlational study, it is still interesting that more societal gender inequalities tend to be observed when the extent of visible linguistic gender asymmetries is higher.

Further, by using masculine-only forms gender stereotypes are being perpetuated and women as well as all other genders except men are being excluded from the discourse (Swim et al., 2004). This restriction of visibility has profound consequences on motivation, self-identification, and one's sense of belonging (Stout and Dasgupta, 2011). Indeed, the use of masculine language formulations tend to result in outcomes that are biased toward men and disadvantage women (Sczesny et al., 2015). This becomes especially evident in studies focusing on the link between the use of gender-fair language and job advertisements, where the display of masculine-only forms makes it harder for women to be considered as suitable for the respective occupation (Horvath and Sczesny, 2015). In addition, gender-fair language seems to be helpful in achieving control over automatically activated cultural gender stereotypes (Kollmayer et al., 2018). Numerous studies have investigated the relationship between language and the mental representations evoked by it (see for example: Stahlberg et al., 2007; Sato et al., 2016). The fact that masculine-only forms are indeed not interpreted as referring to women and other genders but are mainly understood in a male-biased way emphasizes the need to implement gender-fair language exhaustively (Sczesny et al., 2015; Misersky et al., 2019). Research has shown that college students generally display more positive attitudes toward the equality of all genders and are more prone to actually using gender-fair language (Sarrasin et al., 2012). The use of (non) gender-fair language has also been linked to sexist attitudes. To be more precise, participants with sexist beliefs tend to use more gender-exclusive and less gender-fair language (Swim et al., 2004). Correspondingly, the question is being raised how people actually decide to use gender-fair language. Sczesny et al. (2015) showed that the arbitrary and spontaneous use of gender-fair language is actually rather guided by one's deliberate intentions and habits than by one's sexist beliefs. This means that one can actively decide to include gender-fair language in one's behavior. However, critics raise their concern that texts become less comprehensible and less aesthetically appealing when written in gender-fair language (for example: Gabriel et al., 2018).

### 1.3 Text comprehensibility

Since the 1920s, the topic of text comprehensibility has been studied in the scientific discourse (Kintsch and Vipond, 1979). In general, two groups of concepts can be differentiated to assess text comprehensibility (Friedrich and Heise, 2019). The first one regards comprehensibility as an inherent feature of the individual text; to be more precise, high-frequency words, for example, make a text more comprehensible and low-frequency words negatively influence its comprehensibility (Benjamin, 2012). According to this understanding, gender-fair language is likely to be considered as an impairment of the comprehensibility, as for example the gender asterisk extends the sentence's length and prolongs individual words (Gabriel et al., 2018). Another reason might be that feminine forms are “less frequent and therefore less comprehensible,” which results in an increased difficulty to comprehend the text (Friedrich and Heise, 2019, p. 53).

However, text comprehensibility is not only influenced by the text's words, coherence, and syntactical structure, but also by prior knowledge and one's working memory capacity (Schurer et al., 2020). Friedrich (2017) states that general reading skills, for example, play a considerable role, too. Taking this information into account, it can be argued that the view that comprehensibility is solely a characteristic of the text itself is rather insufficient. Instead, Kintsch (1998) as well as McNamara and Magliano (2009) argue that meaning is created in an interaction between the text and the reader. This means that if a particular reader can draw on prior knowledge while reading a specific text, they are more likely to fluently read and process the new information. Linking this interactionist view to one's everyday experience, this concept seems very plausible. This becomes clear in the following example: A book on biological psychology might be hard to understand at the beginning of the semester, but easier to read at the end of it since the state of knowledge has substantially increased.

In addition, an effect on aesthetic appeal can be expected: Friedrich et al. (2021), as well as Friedrich and Heise (2022), argue that comprehensibility is a form of fluency and further, according to fluency theory, stimuli that can be easily processed are evaluated more positively (e.g., Reber and Greifeneder, 2017). One can therefore assume that less complex texts are more aesthetically appealing.

To investigate whether or not gender-fair language in the form of the gender asterisk influences text comprehensibility, several variables such as the ease with which readers can ascribe meaning to the words of a text must be considered. However, there is a lack of research in this area. Especially scarce are studies that explore these effects in adults without an academic background. The use of gender-fair language might be something that is not part of adult non-academics' everyday lives, which would make it rather new and unusual and therefore draw additional attention to itself (Posselt, 2017). This distraction might lead to lower scores in understanding and text comprehensibility.

## 2 Present study

The present study's aim was to test whether the use of gender-fair language impairs text comprehensibility. The development of the research questions and hypotheses was mainly based on the studies by Friedrich and Heise (2019) and Friedrich et al. (2021) who studied the effect of gender-fair language on text comprehensibility in samples of university students. In addition, the study by Pöschko and Prieler (2018) acted as a fundamental basis as these researchers also examined individuals without an academic background, namely vocational students. For this reason, the general comprehensibility of a text, the ease with which readers can ascribe meaning to the words of a text, the ease with which readers can analyse the syntax of the sentences of a text and the aesthetic appeal of texts were investigated. Again, following the example of Friedrich and Heise (2019) and Friedrich et al. (2021), no other aspects of comprehensibility have been tested. Even though the current study situation is rather ambiguous because the two mentioned studies reached different results regarding a possible impairment of text comprehensibility when using gender-fair language, the hypotheses were formulated directionally. To the author's knowledge, the hypotheses have only once been tested on a sample of adult non-academics before (see Rothmund and Christmann, 2002). However, it is the first study to investigate if adults with and without an academic background differ in their ratings of the comprehensibility of texts using different language forms. We therefore proceeded from the following assumptions and tested them in participants with and without an academic background, while also considering differences between these groups:

**Hypothesis 1a:** The use of the gender star reduces the general comprehensibility of texts.
**Hypothesis 1b:** The use of the gender star reduces the ease with which readers can ascribe meaning to the words of a text.
**Hypothesis 1c:** The use of the gender star reduces the ease with which readers can analyse the syntax of the sentences of a text.
**Hypothesis 1d:** The use of the gender star reduces the aesthetic appeal of texts.
**Hypothesis 1e:** A text using the gender star will be evaluated more positively by participants with an academic background than by those without an academic background.

In addition, we examined the participants' attitudes regarding gender-fair language. Swim et al. (2004) have shown that participants' sexist beliefs can be associated with a decrease in the use of gender-fair language. In addition, Sczesny et al. (2015) explored the relationship between the use of gender-fair language and the role of deliberate and habitual factors in predicting it. However, to the author's knowledge, there is no research so far that links subjects' attitudes toward gender-fair language with the effects of gender-fair language on text comprehensibility. The hypotheses that try to close this identified research gap will again be examined in both individuals with and without an academic background, also considering differences between these groups:

**Hypothesis 2a:** Positive attitudes toward the use of gender-fair language are associated with higher comprehensibility of texts written in gender-fair language.
**Hypothesis 2b:** Positive attitudes toward the use of gender-fair language are related to easier ascription of meaning to the words of a text written in gender-fair language.
**Hypothesis 2c:** Positive attitudes toward the use of gender-fair language are associated with greater ease with which readers can analyse the syntax of the sentences of a text written in gender-fair language.
**Hypothesis 2d:** Positive attitudes toward the use of gender-fair language are related to higher aesthetic appeal of texts written in gender-fair language.

## 3 Method

### 3.1 Participants and procedure

Subjects were recruited via three online platforms, namely Facebook, Instagram, and WhatsApp. In addition, participants were asked to recruit further subjects (i.e., snowball sampling). Inspired by Friedrich et al. (2021), they were asked to participate in a study about how people think and feel when they read a text.

The study was conducted in the German language. In sum, 233 participants (49 male, 176 female) voluntarily took part in it. Of the 233 participants, 163 completed the questionnaire in full and were included in the analysis. Their mean age was *M* = 37.63 years (*SD* = 15.38, range = 17–79 years). The sample was divided in two groups, one consisting only of participants who have never studied and are not currently studying at a university or any other higher education institution, and one consisting of participants who are currently studying or have studied in the past. The study's final sample therefore consists of 81 participants (19 male, 62 female) with a mean age of *M* = 44.28 years (*SD* = 14.33, range = 18–68 years) without an academic background and 82 participants (20 male, 61 female, 1 diverse) with a mean age of *M* = 31.32 years (*SD* = 12.36, range = 19–60 years). According to a power analysis carried out with the software G^*^Power for a one-way ANOVA (Faul et al., 2009), a sample size of at least 128 people was necessary to ensure sufficient statistical power of 1–β = 0.80, an α = 0.05 and *d* = 0.50. The effect size was chosen as it is comparable to other studies such as Friedrich and Heise (2022). The majority of adults without an academic background, namely 42.0% (*n* = 34) have passed their A-levels and 23.5% (*n* = 19) graduated from a vocational secondary school without Matura (=school leaving examination). 23.5% (*n* = 19) have an apprenticeship diploma and 8.6% (*n* = 7) have a compulsory school leaving certificate. 2.5% (*n* = 2) do not have a compulsory school leaving certificate. Among participants with an academic background, 31 are currently studying while 51 have completed their studies in the past.

### 3.2 Materials

As material, two versions of a German text on traveling tips on the Spanish island Mallorca were used. The text's theme has been chosen because it can be considered as noncontroversial and easy to understand. The masculine-only forms text consisted of 234 words. An example sentence is as follows: *Weltenbummler, die den einen oder anderen Nervenkitzel im Urlaub möchten, sind auf Mallorca genau richtig*. (“For globetrotters who want a thrill or two on holiday, Mallorca is the place to be.”). In order to create a gender-fair version of said text, all 10 masculine-only forms were systematically replaced with gender asterisk forms, e.g., *Weltenbummler* (globetrotters) with Weltenbummler^*^innen. The texts as well as the questionnaire in full length can be found in the repository Open Science Framework (https://osf.io/).

### 3.3 Measures

The study had two factors, namely (1) the text type which is being expressed by either belonging to the condition only using masculine forms or to the condition using gender-fair language by applying the gender asterisk and (2) the academic background.

The dependent variables are as follows: overall subjective comprehensibility (Cronbach's α = 0.87), word difficulty (Cronbach's α = 0.80), sentence difficulty (Cronbach's α = 0.85) and aesthetic appeal (Cronbach's α = 0.88). With internal consistencies between Cronbach's α = 0.80 and 0.95, the scales reliabilities proved to be acceptable or excellent. The first four dependent variables–which are subjective comprehensibility, word difficulty, sentence difficulty and aesthetic appeal–were assessed using Friedrich's (2017) questionnaire on comprehensibility. Each of the factors is being measured using five items. However, only the four subscales for which hypotheses had been formulated were used for the present project. All of the scales are answered on a 5-point Likert scale ranging from 1 = “I disagree” to 5 = “I agree.” An example item for the *overall subjective comprehensibility* is “I thought the text was comprehensible” (*Ich fand den Text verständlich*). The ease of ascribing meaning to words was measured using the scale *word difficulty*, one item being “For some words, I was not sure what they meant” (*Bei einigen Wörtern war ich mir nicht sicher, was sie bedeuten*). The ease of decoding the syntactical structure of the text's sentences was measured by the scale *sentence difficulty*, for example by the item “The sentences had a complicated structure” (*Die Sätze hatten eine komplizierte Struktur*). Finally, the text's aesthetic appeal to the readers was measured by the scale *variety of language use*, a sample item being “I found the language lively” (*Ich fand die Sprache lebhaft*). Items have been (re)coded so that higher values indicate better comprehensibility, lower word difficulty, lower sentence difficulty and higher aesthetic appeal.

In order to operationalise the last dependent variable, namely the participants' attitudes toward gender-fair language, a questionnaire on the participants' attitudes toward gender-fair language by Sczesny et al. (2015) who adopted the items from earlier studies (e.g., Knussen et al., 2004) was used. It includes nine items that can be reliably combined into a single scale (Cronbach's α = 0.95). The items are answered on a 7-point Likert scale ranging from 1 = “strongly disagree” to 7 = “strongly agree.” One sample item is as follows: “It is personally important to me to use gender-inclusive language” (*Es ist mir persönlich wichtig, eine geschlechtergerechte Sprache zu verwenden*).

### 3.4 Procedure

The study used a between-subjects design with the factor language form (masculine-only vs. gender-fair language). As already mentioned, the study was conducted using an online questionnaire. Altogether, the experiment was divided in five large sections: After having read the instructions and providing informed consent (1), the participants' demographics–including gender, age, and highest education–was assessed (2). Next, the participants read one of the two randomly assigned versions of the text (3). Subsequently, comprehensibility scales followed (4) and lastly, the participants answered questions about their attitudes regarding gender-fair language (5). Participants could not go back from the questionnaire about their attitudes toward gender-fair language to the earlier parts of the study. The questionnaire has been designed using SoSci (https://www.soscisurvey.at/) (Leiner, 2022). Filling in the questionnaire took around 6 min (*M* = 362.49 sec, *SD* = 117.76).

Participation was anonymous, and all data has been kept absolutely confidential. Moreover, participation was voluntary, and the subjects were not identifiable. The participants have been told that there were no right or wrong answers and that they could withdraw from the study at any time. Lastly, they confirmed that they have been informed about the conditions of participation and that they would like to take part in the study.

### 3.5 Statistical analysis

For data analysis and answering the research questions, descriptive statistics were determined with IBM SPSS Statistics 28 and a multivariate, multifactorial analysis of variance was carried out as were bivariate correlation analyses. The α-error level was set at 5% for all calculation. According to Cohen (1992), the effect size $\eta_{\text{p}}^{2}$ (partial eta squared) was classified as follows: $\eta_{\text{p}}^{2}$ of 0.01 were considered as small, $\eta_{\text{p}}^{2}$ of 0.06 as medium and $\eta_{\text{p}}^{2}$ of 0.14 as large. In case of correlations, *r* between 0.1 and 0.3 are interpreted as small to moderate, *r* between 0.3 and 0.5 as moderate to large and from *r* = 0.5 as large.

## 4 Results

In order to test the first set of hypotheses, a multivariate analysis of variance (MANOVA) with the two independent variables “text version” “(gender star vs. masculine-only forms)” and “group (academic background vs. no academic background” has been conducted to find out whether who had read a text in GFL perceived the text as harder to comprehend than those who read the text in masculine-only forms, and if participants academic background played a role for perceived text comprehensibility. Since multicollinearity may have emerged, a correlational analysis of the four facets of text comprehensibility was carried out first. Bivariate correlations between dependent variables were well below *r* < 0.90, indicating that multicollinearity was not a confounding factor in the analysis (Field, 2017). Table 1 shows the correlation matrix.

**Table 1:** Correlation matrix of the pre-analysis of the MANOVA.

	**1**.	**2**.	**3**.	**4**.
1. Subj. comprehensibility	-	-	-	-
2. Word difficulty	0.47^**^	-	-	-
3. Sentence difficulty	0.72^**^	0.53^**^	-	-
4. Aesthetic appeal	0.45^**^	0.26^**^	0.46^**^	-

^**^Correlation is significant at the 0.01 level (2-tailed).

There was no statistically significant multivariate main effect of the text version on comprehensibility, *F*_(4, 156)_ = 0.27, *p* = 0.90, $\eta_{\text{p}}^{2}$ = 0.01. Contrary to expectations, the use of gender-fair language did not reduce the general subjective comprehensibility of texts as no statistically significant effect on comprehensibility could be found, *F*_(1, 159)_ = 0.56, *p* = 0.81, $\eta_{\text{p}}^{2}$ = 0.00. Similarly, there was no statistically significant effect on word difficulty, *F*_(1, 159)_ = 0.17, *p* = 0.90, $\eta_{\text{p}}^{2}$ = 0.00. The use of gender-fair language did not significantly reduce the ease with which readers can analyse the syntax of the sentences of a text, *F*_(1, 159)_ = 0.70, *p* = 0.40, $\eta_{\text{p}}^{2}$ = 0.00. Regarding the question if the use of gender-fair language reduces the aesthetic appeal of texts, no statistically significant effect could be detected, *F*_(1, 159)_ = 1.84, *p* = 0.67, $\eta_{\text{p}}^{2}$ = 0.00. This means that comprehensibility was not significantly dependent on which prior text the participants had read.

We found a significant multivariate main effect of participants' academic background on comprehensibility, *F*_(4, 156)_ = 2.88, *p* = 0.02, $\eta_{\text{p}}^{2}$ = 0.07. The only significant univariate difference occurred in the aesthetic appeal of texts, which was assessed significantly higher by participants without an academic background, *F*_(1, 159)_ = 6.48, *p* = 0.01, $\eta_{\text{p}}^{2}$ = 0.04. There were no significant differences in comprehensibility, *F*_(1, 159)_ = 0.30, *p* = 0.86, $\eta_{\text{p}}^{2}$ = 0.00, word difficulty, *F*_(1, 159)_ = 0.37, *p* = 0.87, $\eta_{\text{p}}^{2}$ = 0.00, and sentence difficulty, *F*_(1, 159)_ = 3.20, *p* = 0.08, $\eta_{\text{p}}^{2}$ = 0.02.

There was no multivariate significant interaction effect of text version and academic background on comprehensibility, *F*_(4, 156)_ = 0.49, *p* = 0.75, $\eta_{\text{p}}^{2}$ = 0.01, indicating no joint effects of these two variables, *F*_(1, 159)_ = 1.00, *p* = 0.32, $\eta_{\text{p}}^{2}$ = 0.01, word difficulty, *F*_(1, 159)_ = 1.34, *p* = 0.25, $\eta_{\text{p}}^{2}$ = 0.01, sentence difficulty, *F*_(1, 159)_ = 1.47, *p* = 0.23, $\eta_{\text{p}}^{2}$ = 0.01, or aesthetic appeal, *F*_(1, 159)_ = 0.07, *p* = 0.80, $\eta_{\text{p}}^{2}$ = 0.00.

The descriptive statistics of the dependent variables by language condition and participants' academic background can be found in Table 2.

**Table 2:** Descriptive statistics of subjective comprehensibility, word difficulty, sentence difficulty, and aesthetic appeal by language condition and participants' academic background.

	**Academic background**	**Language condition**	**M**	**SD**	***n***
Subj. comprehensibility	Yes	GFL	4.41	0.62	35
		MOF	4.31	0.85	47
		Total	4.36	0.76	82
	No	GFL	4.31	0.95	43
		MOF	4.46	0.68	38
		Total	4.38	0.83	81
	Total	GFL	4.35	0.82	78
		MOF	4.38	0.78	85
		Total	4.37	0.80	163
Word difficulty	Yes	GFL	4.27	0.86	35
		MOF	4.14	0.77	47
		Total	4.19	0.81	82
	No	GFL	4.14	0.83	43
		MOF	4.31	0.83	38
		Total	4.22	0.83	81
	Total	GFL	4.20	0.84	78
		MOF	4.21	0.80	85
		Total	4.21	0.82	163
Sentence difficulty	Yes	GFL	4.09	0.73	35
		MOF	4.04	0.82	47
		Total	4.06	0.78	82
	No	GFL	4.16	0.88	43
		MOF	4.42	0.73	38
		Total	4.28	0.82	81
	Total	GFL	4.13	0.81	78
		MOF	4.21	0.80	85
		Total	4.17	0.80	163
Aesthetic appeal	Yes	GFL	3.69	0.83	35
		MOF	3.71	0.98	47
		Total	3.70	0.91	82
	No	GFL	4.01	0.86	43
		MOF	4.11	0.83	38
		Total	4.05	0.84	81
	Total	GFL	3.87	0.86	78
		MOF	3.89	0.93	85
		Total	3.88	0.89	163

GFL, gender-fair language; MOF, masculine-only forms.

In order to test the second set of hypotheses, we conducted bivariate correlation analyses between attitudes toward the use of gender-fair language and the four facets of text comprehensibility in participants with and without an academic background. In both conditions, the different aspects of text comprehensibility were significantly interrelated, which is reflected in the correlation coefficients in Table 3. More positive attitudes toward the use of GFL were not associated with higher ratings of text comprehensibility in both conditions.

**Table 3:** Correlation matrix of attitudes toward the use of gender-fair language and text comprehensibility scales for both language conditions.

	**1**.	**2**.	**3**.	**4**.	**5**.
1. Attitudes toward GFL	-	0.13	0.04	−0.02	0.02
2. Subj. comprehensibility	−0.20	-	0.52^**^	0.77^**^	0.54^**^
3. Word difficulty	−0.12	0.42^**^	-	0.58^**^	0.30^**^
4. Sentence difficulty	−0.25^*^	0.66^**^	0.48^**^	-	0.60^**^
5. Aesthetic appeal	−0.22	0.32^**^	0.22^*^	0.34^**^	-

GFL, gender-fair language; correlation coefficients for participants having read the text in masculine-only forms are reported below the diagonal; participants heaving read the text in GFL, above the diagonal. ^**^Correlation is significant at the 0.01 level (2-tailed). ^*^Correlation is significant at the 0.05 level (2-tailed).

## 5 Discussion

Overall, studies on gender-fair language are important for promoting greater inclusivity, representation, accuracy, and progress toward gender equality. In line with this, the aim of the present study was to examine if the use of gender-fair language in the form of the gender asterisk makes texts less comprehensible than the use of masculine-only forms. To the authors' knowledge, this has been the first experiment in the field of text comprehensibility and gender-fair language that has examined the effect of the academic background. As mentioned above, this target group had so far only been investigated extremely rarely, resulting in the urgent need to close said identified research gap.

According to the results of the present study and contrary to the often-repeated claims of critics of gender-fair language (Vergoossen et al., 2020), the gender asterisk did not impair subjective comprehensibility, the ease of ascribing meaning to the text's words, the ease of decoding the syntax of the sentences, or the aesthetic appeal of the text in any statistically significant manner. In line with previous research, these different aspects of text comprehensibility were related to each other. The participants did not have problems understanding the texts in gender-fair language, which were produced by removing words that are exclusively masculine and instead substituting with gender-neutral language forms using the gender asterisk. This was not only true for participants with an academic background but also for participants who never studied at a higher education institution. Moreover, the participants' attitudes toward gender-fair language did not statistically influence text comprehensibility in participants who had read a text in gender-fair language.

In general, previous studies indicate that pair forms (Braun et al., 2007; Friedrich and Heise, 2019), neutral forms (Rothmund and Christmann, 2002; Steiger-Loerbroks and von Stockhausen, 2014), capital-I forms (Klimmt et al., 2008; Pöschko and Prieler, 2018), or gender asterisks in plural forms (Friedrich et al., 2021) do not negatively affect comprehensibility, but gender asterisks in singular forms and slashes do (Friedrich et al., 2021). As the present study mainly used plural forms in combination with the gender asterisk, the findings seem to be in line with the previous research as no problems with comprehensibility occurred although we used the gender asterisk not only in plural forms but also in singular forms. While our research design does not allow us to distinguish whether the singular forms were harder to understand than the plural forms, we see no general impairment of the text's comprehensibility in the GFL condition. This could be due to the fact that in contrast to complete pair forms (Lehrerinnen und Lehrer; “teachers_fem_” and “teachers_masc_”) the gender asterisk does not lead to noteworthy longer sentences. Moreover, nouns using the gender asterisk may still be recognizable enough not to count as low-frequency words in terms of comprehensibility. In future studies, it is increasingly important to investigate the comprehensibility of singular forms. In order to compare the influence of singular and plural forms on language use, future studies could employ single-factor designs that have three factor levels: the gender asterisk in plural forms, the gender asterisk in singular forms, and the masculine-only forms. Furthermore, studies show that texts written in gender-fair language have less aesthetic appeal than texts written in masculine-only language (Rothmund and Christmann, 2002; Klimmt et al., 2008). However, this finding could not be supported by the present study. Instead, the gender asterisk did not appear to have any impairing effects on aesthetic appeal.

A possible explanation for the study's results might be the low level of complexity of the given text. As Friedrich and Heise (2019) pointed out, the overall language and structure of texts is a highly relevant predictor of comprehensibility. Future studies could therefore investigate whether there is an effect of gender-fair language when using complex syntactic structures.

Proceeding with the second package of hypotheses, contrary to expectations, negative attitudes toward the use of gender-fair language were not associated with lower subjective comprehensibility, a more difficult ascription of meaning to the words of a text written in gender-fair language, a diminished ease with which readers can analyze the syntax of the sentences of a text written in gender-fair language nor with lower aesthetic appeal of texts written in gender-fair language. These findings further strengthen the argument that gender-fair language does indeed not impair comprehensibility, not even in individuals who reject GFL.

The study's results, though, are subject to a number of limitations. First of all, the composition of the sample is not balanced as the large majority of the participants is female. This is a well-known phenomenon as women tend to respond in greater proportions to online questionnaires than men (Porter and Whitcomb, 2005). Additionally, there is an underrepresentation of genders other than male and female. It would be highly interesting to account for these problems in future studies. Further, no analysis of reading times has been carried out. It is quite possible that reading gender-fair texts might take the participants more time as they are not used to reading such texts. This is why the results should be replicated with further measures.

Last and maybe most important, the product of comprehension was not assessed. To evaluate comprehension, the participants would have needed to be given tasks that require them to use the text for answers but also go beyond the text, allowing for responses that can be judged as either correct or incorrect (see, for example, Schnotz, 1994; Kintsch, 1998).

Summa summarum, the results of the present study are overall consistent with those of previous research, but explicitly extend them to individuals who are less attuned to reading GFL than university students and academics. As gender-fair language use did not affect subjective text comprehensibility regardless of educational background, it can be concluded that the widespread argument against its use can simply not be supported.

## Data availability statement

The datasets presented in this study can be found in online repositories. The names of the repository/repositories and accession number(s) can be found below: https://osf.io/agvy6/?view_only=8e4694e948c649a3acd036531709c6f7.

## Ethics statement

Ethical approval was not required for the studies involving humans because of the non-invasive, voluntary nature of the study with no anticipated negative consequences. The studies were conducted in accordance with the local legislation and institutional requirements. The participants provided their written informed consent to participate in this study.

## Author contributions

LP and MK developed the study concept. LP prepared the draft manuscript and MK provided critical revisions. All authors contributed meaningfully to the paper. All authors approved the final version of the manuscript for submission.
